# Cytokine and Nitric Oxide Levels in Patients with Sepsis – Temporal Evolvement and Relation to Platelet Mitochondrial Respiratory Function

**DOI:** 10.1371/journal.pone.0097673

**Published:** 2014-05-14

**Authors:** Fredrik Sjövall, Saori Morota, Eleonor Åsander Frostner, Magnus J. Hansson, Eskil Elmér

**Affiliations:** 1 Mitochondrial Medicine, Department of Clinical Sciences, Lund University, Lund, Sweden; 2 Copenhagen University Hospital, Rigshospitalet, Intensive Care Unit 4131, Copenhagen, Denmark; 3 Department of Clinical Physiology, Skåne University Hospital, Lund University, Lund, Sweden; 4 Department of Clinical Neurophysiology, Skåne University Hospital, Lund University, Lund, Sweden; Fundação Oswaldo Cruz, Brazil

## Abstract

**Background:**

The levels of nitric oxide (NO) and various cytokines are known to be increased during sepsis. These signaling molecules could potentially act as regulators and underlie the enhancement of mitochondrial function described in the later phase of sepsis. Therefore, we investigated the correlation between observed changes in platelet mitochondrial respiration and a set of pro- and anti-inflammatory cytokines as well as NO plasma levels in patients with sepsis.

**Methods and Results:**

Platelet mitochondrial respiration and levels of TNFα, MCP-1 (monocyte chemotactic protein-1), INFγ (interferon-γ), IL-1β, IL-4, IL-5, IL-6, IL-8, IL-10 and IL-17 and NO were analyzed in 38 patients with severe sepsis or septic shock at three time points during one week following admission to the ICU. Citrate synthase, mitochondrial DNA and cytochrome *c* were measured as markers of cellular mitochondrial content. All mitochondrial respiratory states increased over the week analyzed (p<0.001). IL-8 levels correlated with maximal mitochondrial respiration on day 6–7 (p = 0.02, r^2^ = 0.22) and was also higher in non-survivors compared to survivors on day 3–4 and day 6–7 (p = 0.03 respectively). Neither NO nor any of the other cytokines measured correlated with respiration or mortality. Cytochrome *c* levels were decreased at day 1–2 by 24±5% (p = 0.03) and returned towards values of the controls at the last two time points. Citrate synthase activity and mitochondrial DNA levels were similar to controls and remained constant throughout the week.

**Conclusions:**

Out of ten analyzed cytokines and nitric oxide, IL-8 correlated with the observed increase in mitochondrial respiration. This suggests that cytokines as well as NO do not play a prominent role in the regulation of platelet mitochondrial respiration in sepsis. Further, the respiratory increase was not accompanied by an increase in markers of mitochondrial content, suggesting a possible role for post-translational enhancement of mitochondrial respiration rather than augmented mitochondrial mass.

## Introduction

Despite extensive research, sepsis continues to be a devastating disease with high mortality, primarily due to unresolving multiple organ failure (MOF) [1]. The pathogenesis of, and recovery from, MOF has also been studied extensively but remains an enigma, much due to its complexity [2].

Mitochondrial dysfunction has been implicated in the pathogenesis of sepsis [3], [4] and although most results point toward an initial dysfunction of mitochondrial respiration the exact mechanisms are not clear and results are still conflicting. The same events triggering an impaired function in the initial phases of sepsis are also signals for the increase in energy demand required and stimulate mitochondrial biogenesis, *e.g.* the regeneration of mitochondria within the cell. Mitochondrial biogenesis and increased mitochondrial respiratory capacity have been demonstrated in animal models and in humans in the later stages of the septic process [5]–[8].

Only 13 of the essential respiratory complex subunits are encoded by the mitochondrial DNA (mtDNA). Hence, mitochondrial biogenesis is dependent on protein synthesis derived from transcription and translation of both mitochondrial and nuclear DNA [9]. PGC-1α (peroxisome proliferator-activated receptor gamma (PPARγ) co-activator 1-α) has been demonstrated as a master regulatory protein for mitochondrial biogenesis via co-activation of a variety of transcription factors such as nuclear respiratory factor 1 and 2 (NRF-1 and NRF-2) and mitochondrial transcription factor A (TFAM) [10]–[12]. The expression of PGC-1α is in turn modulated by a variety of stimuli such as cold, fasting, exercise and inflammation that acts through cellular signaling systems [11], [13].

In sepsis, production of cytokines is greatly upregulated and released by cells from the innate and adaptive immune system to modulate the inflammatory response [14]–[16]. Also, increased nitric oxide (NO) production from the upregulation of inducible nitric oxide synthase (iNOS) is characteristic of sepsis [17], [18].

Cytokines, such as IL-1β and TNFα, and NO have been shown to activate PGC-1α by phosphorylation and a cGMP signaling pathway, respectively, and as such serve as a physiological stimuli of mitochondrial biogenesis [19]–[22]. NF-κB, another transcription factor central for the regulation of inflammation, has also recently been implicated as a regulator of mitochondrial oxidative phosphorylation [23]. In light of these findings we hypothesized that cytokine and NO levels in plasma from septic patients would correlate with the increase in mitochondrial respiration during the first week of sepsis that we have previously demonstrated in platelets [8].

## Material and Methods

### Patients and ethics statement

Adult (>18 years) patients with severe sepsis or septic shock, as previously defined [24], were recruited between august 2008 to September 2011 from the intensive care units (ICU) of Lund University Hospital and Copenhagen University Hospital, Rigshospitalet. Controls were recruited in Lund, Sweden from healthy volunteers. The study was carried out in compliance with national legislation and the Code of Ethical Principles for Medical Research Involving Human Subjects of the World Medical Association (Declaration of Helsinki). The study was approved by the scientific ethical committee of Copenhagen county, Denmark (H-C-2008-023) and the regional ethical review board of Lund, Sweden (113/2008, 2011/79 and 2011/89). Samples were taken after written informed consent was acquired from control subject, patient or next of kin. In Denmark, for patients deemed temporarily mental incompetent, written informed consent was acquired from next of kin as well as from the patient's primary health care physician as requested by current legislation. Patients were included within 48 h after their admission to the ICU. Patients that were pregnant, had a known mitochondrial disease or hematological malignancy were excluded.

### Sample preparation

Blood were sampled from an arterial line in patients and by venous puncture in controls and collected in K_2_EDTA tubes (Vacuette, Greiner Bio-One GmbH, Kremmünster, Austria). Patients were sampled three times during the first week at the ICU; within the first 48 h (day 1–2), on day 3–4 and day 6–7. Platelet preparation was commenced immediately after sampling. The vials were centrifuged at 300×*g* 15 min. The supernatant, platelet rich plasma, was collected and centrifuged at 4600×*g* for 5 min. The ensuing pellet was suspended in a small amount of plasma with a final platelet concentration of approximately 2000×10^9^/L. Respirometric measurements were performed directly after isolation and within 5 hours of sampling. The analyzed contents from the respirometry chamber were stored frozen until further use.

For incubation experiments, platelets were isolated from buffy coat collected from healthy donors the same day. The buffy coat was divided into K_2_EDTA vials and platelets were isolated as described above. The platelets were then resuspended in either healthy donor plasma or plasma from septic patients.

### Determination of mitochondrial oxygen consumption by high-resolution respirometry

Respiration was measured at 37°C in 2 ml glass chambers using a high-resolution oxygraph with online display of the calibrated oxygen concentration and oxygen flux, *i.e.* the negative time derivative of oxygen concentration (OROBOROS Oxygraph-2k and DatLab software, OROBOROS, Instruments, Innsbruck, Austria). Calibration with air-saturated Millipore water was performed daily. The oxygen concentration was calculated from the digitally recorded barometric pressure and the oxygen solubility at 37°C. The oxygen solubility factor relative to pure water was set to 0.92 for MiR05. Oxygen consumption was expressed as pmol/s/10^6^ cells.

Platelets at a concentration of 50–200×10^6^/ml were suspend in respiration medium consisting of 110 mM sucrose, 0.5 mM EGTA, 3.0 mM MgCl_2_, 80 mM KCl, 60 mM K-lactobionate, 10 mM H2PO_4_, 20 mM taurine, 20 mM HEPES and 1.0 g/l BSA, pH 7.1 (MiR05) [25]. Oxidative capacities (OXPHOS) were determined in the presence of saturating concentrations of oxygen and ADP (1 mM) using a substrate, inhibitor titration (SUIT) protocol as described previously [8]. Platelets were permeabilized with digitonin (1 µg/1×10^6^ platelets). For complex I-dependent respiration (OXPHOS_CI_), substrates were pyruvate (5 mM) plus malate (5 mM) and glutamate (5 mM) which provide nicotinamide adenine dinucleotide (NADH) to the respiratory chain. Maximal OXPHOS is obtained by convergent electron input through both complex I and complex II (OXPHOS_CI+II_) and was determined by sequentially adding succinate (10 mM). State 4 (with CI and CII substrates present, LEAK) was evaluated by adding oligomycin (1 µg/ml) and maximal capacity of the electron transport system (ETS_CI+II_) was further obtained by careful titration of the protonophore, carbonyl cyanide p-(trifluoromethoxy) phenylhydrazone (FCCP). For measurement of complex II-dependent respiration (ETS_CII_) complex I was subsequently inhibited by rotenone (2 µM). Electron flow through complex I to III was inhibited by addition of antimycin-A, and the residual oxygen consumption was subtracted from prior oxidative values. Complex IV-dependent respiration (CIV) was measured by adding N,N,N′,N′-tetramethyl-p-phenylendiamine (TMPD, 0.5 mM). As TMPD exhibited a wide range of auto-oxidation in the sample preparation, respiration was finally inhibited with sodium azide (10 mM) and the difference between the oxygen consumption before and after the addition of sodium azide was determined as complex IV respiration. The coupling of phosphorylation to oxidation was determined by calculating control ratios for both maximal capacity of OXPHOS and ETS by dividing the respective rate with state 4 respiration. For experiments on intact cells, platelets were incubated in their own plasma and allowed to stabilize at routine level. Subsequent addition of oligomycin induced State 4 (LEAK) and FCCP was thereafter titrated until maximal respiration was achieved. Addition of rotenone and antimycin-A ended the experiment and residual oxygen consumption was subtracted from prior respiration values.

### Cytokine measurement

Cytokines were analyzed with a multiplex sandwich immunoassay format with electrochemiluminescence according to manufacturer's instructions (MSD 96-well Multi-Spot, Meso Scale Discovery, Gaithersburg, Maryland, USA). In short, 96-well plates pre-coated with capture antibodies for TNFα, MCP-1 (monocyte chemotactic protein-1), INFγ (interferon-γ), IL-1β, IL-4, IL-5, IL-6, IL-8, IL-10 and IL-17 were incubated with plasma samples for 2 hours. Subsequently, detection antibodies were added and the plate incubated for another 2 hours. After washing, the plate was read with MSD Sector Imager. Since the variation of cytokine levels in the healthy controls was low the sampling was restricted to 12 randomly selected subjects.

### Nitrate + Nitrite measurements

NO levels in plasma are short lived and were thus evaluated by its stable metabolites nitrate (NO_3_) and nitrite (NO_2_) using the Griess reaction. A commercial kit was used with modifications adapted for plasma samples [26] (Sigma 23479, nitrate/nitrite Assay Kit Colorimetric, Sigma-Aldrich, St. Louis, MO, USA). Briefly, samples were incubated with nitrate reductase to reduce nitrate to nitrite. Griess reagent was then added and absorbance was measured at 570 nm to evaluate total nitrite levels (Bio-Rad 680 microplate reader, Bio-Rad Laboratories, CA, USA). Background absorbance was measured for each sample and subtracted from total values.

### Analysis of mitochondrial DNA

Mitochondrial DNA (mtDNA) was measured as previously described [8] with modification. In brief, frozen samples were thawed, sonicated and subsequently diluted 500 times in a buffer containing 10 mM TRIS-HCl, 1 mM EDTA, salmon sperm DNA 1 ng/µl, pH 8.0. PCR reaction was performed in a StepOnePlus Real-Time PCR System (Applied Biosystems Inc., Foster City, CA, USA) with primers targeting the human mitochondrial COX-1 gene (forward: CCC CTG CCA TAA CCC AAT ACC A, reverse: CCA GCA GCT AGG ACT GGG AGA GA). Samples were analyzed in duplicates.

### Cytochrome c determination

Human cytochrome *c* (Cyt *c*) content was quantified using an immunoassay kit (DCTC0, Quantikine, R&D systems, Abingdon, UK). Frozen samples where thawed, sonicated and subsequently processed according to the manufacturer's instructions.

### Citrate synthase determination

A commercially available kit (Citrate Synthase Assay Kit, CS 0720, Sigma), was used according to manufacturer's instructions, to determine citrate synthase (CS) activity in the frozen samples.

### Plasma incubation experiments

Platelets were incubated in 1 ml of plasma from septic patients or healthy volunteers for 1 hour in room temperature before experiments. Mitochondrial respiration was then assessed with the protocol for intact cells described above.

### IL-8 incubation experiments

Blood from healthy volunteers was collected in K_2_EDTA tubes. IL-8 was added to a final concentration of 10 ng/ml and tubes were placed on a tilting board in room temperature for 24 h. Platelets were then isolated and analysed as described above.

### Statistical analysis

Data were tested for normal distribution with D'Agostino and Pearson omnibus normality test. Parametric data are presented as mean ± SD and non-parametric data as median ± interquartile range (IQR). Differences between controls and patients at different time points and plasma incubation experiments were analyzed with ANOVA with post-analysis using Dunnett's Multiple Comparison Test for parametric data and Kruskal-Wallis test with post-analysis using Dunn's Multiple Comparison Test for non-parametric data. Differences between first and last time points in the patient cohort were analysed with paired Student *t*-test. Correlations were evaluated using linear regression. A p-value of <0.05 was considered significant. Statistical analysis was performed using GraphPad Prism 5.04 software (GraphPad Software, Inc, La Jolla, CA, USA).

## Results

Demographics of the 38 patients and 38 controls recruited are presented in Table 1. Five patients died and another 6 patients were discharged or transferred to other facilities within the first week lending the patient cohort at day 3–4 with *n* = 31 and *n* = 27 at day 6–7.

**Table 1:** Demographics of patients and controls.

	Patients	Controls
Age, years	70 (60–74)	67 (57–72)
Sex, male/female (%)	21/17 (55/45)	15/23 (39/61)
SAPS II	48 (40–55)	
APACHE II	23 (17–27)	
SOFA, Day of first sampling	9 (7–11)	
SOFA, Day of second sampling	9 (4–11)	
SOFA, Day of third sampling	6.5 (5–9)	
Source of sepsis		
- Soft tissue	12	
- Abdominal	12	
- Chest	10	
- Blood	2	
- Urinary tract	1	
- Mediastinum	1	
Severe Sepsis/Septic Shock (%)	4/34 (11/89)	
Outcome, number (%)		
28-day mortality	8 (21)	
90-day mortality	13 (34)	

Displayed as median (IQR) or individual numbers (%) SAPS, Simplified Acute Physiologic Score; APACHE, acute physiology and chronic health evaluation score; SOFA, Sequential Organ Failure Assessment.

### Respiration levels

**Figure 1:**
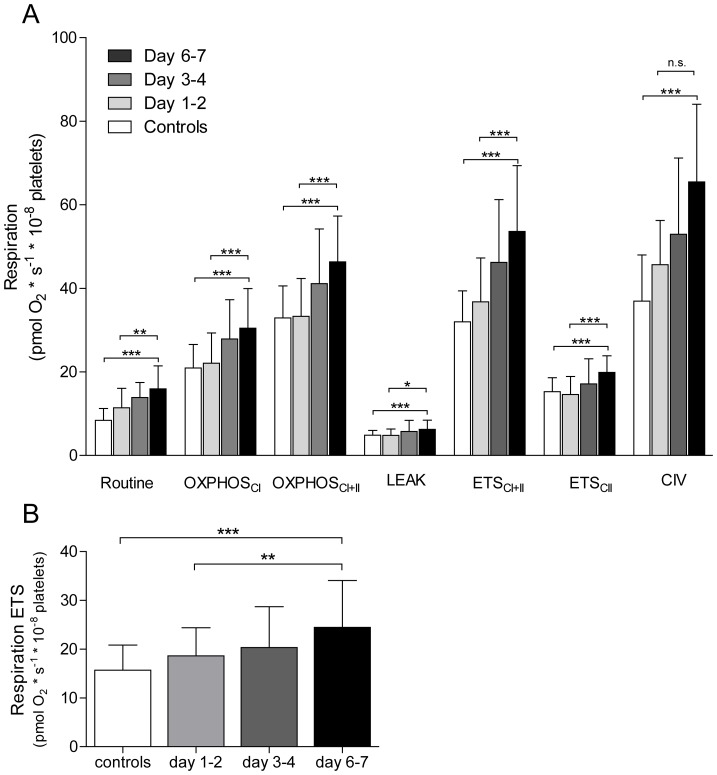
Mitochondrial respiration. Different respiratory states in controls and at three time points over one week in patients with sepsis in permeabilised (A) and intact cells incubated in their own plasma (B). Controls n = 38, patients n, 1st time point = 38, 2nd time point = 31, 3rd time point = 27. Displayed as mean ± SD. * = p<0.05, ** = p<0.01, *** = p<0.001.

In Fig. 1 the levels of mitochondrial respiration in permeabilised (A) and intact (B) cells are displayed. The respiratory data from the first 18 patients has been described earlier [8]. All respiratory parameters gradually increased during the first week of sepsis and were significantly elevated compared to controls at the second (except for CIV) and third time points measured. At day 6–7 respiration of septic patients and controls were as follows: routine 16±5.5 vs. 8.4±2.8, OXPHOS_CI_ 30±9.5 vs. 21±5.6, OXPHOS_CI+II_ 46±11 vs. 33±7.7, LEAK 6.2±2.2 vs. 4.9±1. ETS_CI+II_ 54±16 vs. 32±7.4 and ETS_CII_ 20±4.0 vs. 15±3.3, CIV 65±19 vs. 37±11 and intact cells 24±9.6 vs. 16±5.1 pmol O_2_×s^−1^×10^−8^ platelets, p<0.001. Also, the respiratory control ratio (ETS_CI+II_/LEAK) was significantly increased in the septic patients compared to controls (8.9±2.2 vs 7.8±1.5, p<0.05).

### Cytokine levels

**Figure 2:**
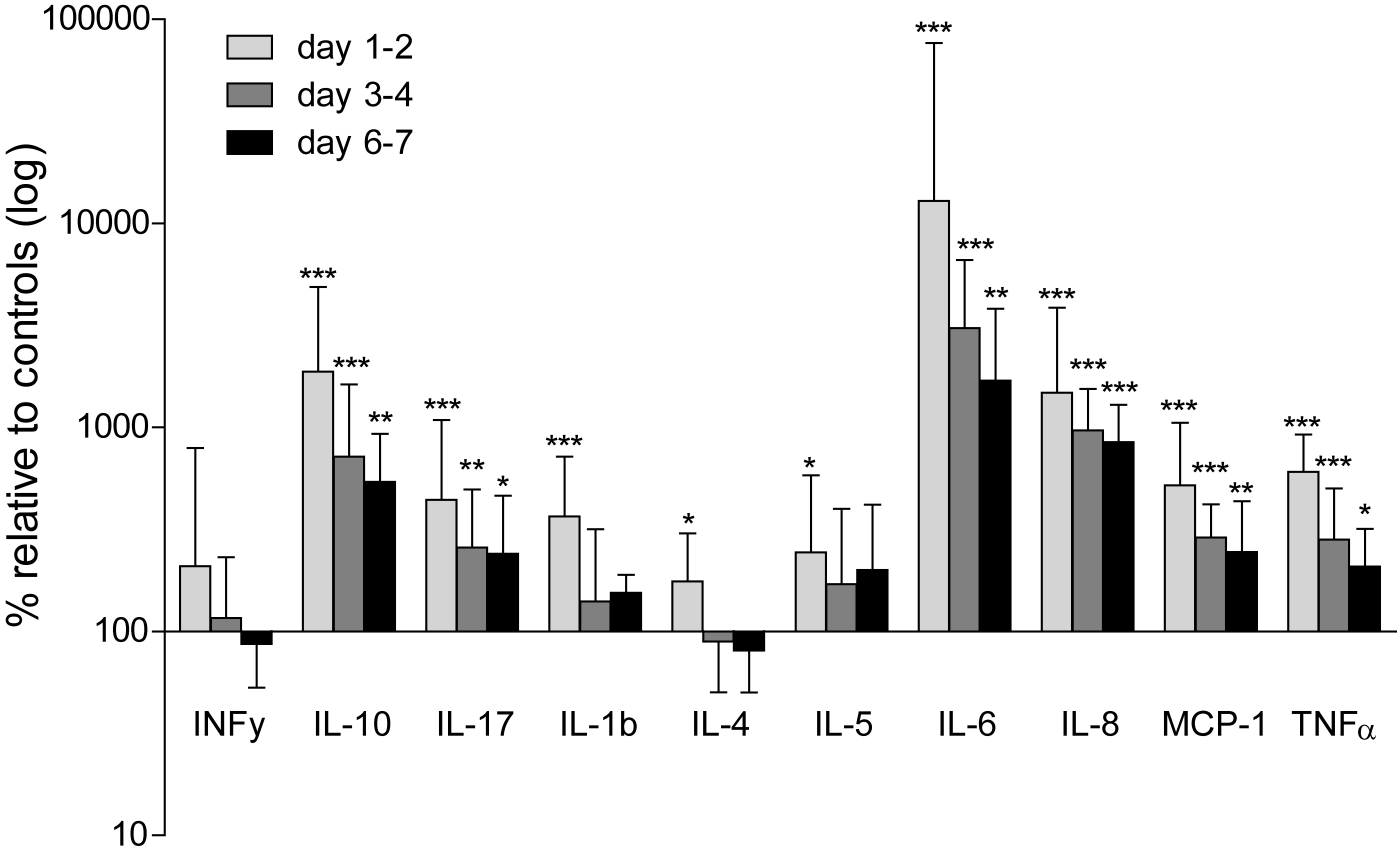
Multiplex cytokines assay. Plasma analysed from septic patients at three different time points (within 48 h, day 3–4 and day 6–7 of ICU stay). Controls were set to 100% for each cytokine and septic plasma values are expressed as log (%) relative difference. Controls n = 12, patients n, 1st time point = 38, 2nd time point = 31, 3rd time point  = 27, bars  =  median ± interquartile range. * = p<0.05, ** = p<0.01, *** = p<0.001 compared to controls.

Of the 10 cytokines measured, plasma concentrations of all, except for INFγ, were significantly increased at the first sampled time point as compared to controls (IL1β 1.9 (0.8–3.7) vs. 0.45 (0.2–0.6), IL-4 1.5 (0.8–2.7) vs. 0.6 (0.3–1.5), IL-5 2.6 (0.9–6.2) vs. 0.9 (0.7–1.3), IL-6 228 (115–1351) vs. 1.7 (1.0–2.3), IL-8 40 (20–104) vs. 2.4 (1.9–3.5), IL-10 26 (11–69) vs. 1.5 (0.9–1.9), MCP-1 1457 (720–2954) vs. 256 (181–382) TNFα 23 (9.0–35) vs. 3.7 (2.7–4.3) pg/ml, p<0.001 except IL-5 p = 0.03). At the subsequent time points IL-6, IL-8, IL-10, IL-17, MCP-1 and TNFα remained significantly elevated compared to controls (30 (18–67), 23 (13–35), 7.7 (4.0–13), 16 (8.8–31), 688 (473–1217) and 7.8 (5.7–12) pg/ml respectively, p<0.01 except IL-17 and TNFα p<0.05) whereas IL-1β, IL-4, IL-5 returned to values similar to controls. The relative changes in cytokine levels as compared to controls are displayed in Fig. 2.

### Nitrate + Nitrite levels

Mean plasma NO_3_+NO_2_ levels were 14.5±3.2 µM in controls. In the septic patients NO_2_+NO_3_ levels were not significantly increased (39.3±8.3 µM, p = 0.2) within 48 h of admission to the ICU, and returned toward control values at the subsequent time points (24.5±6.0 µM at day 3–4 and 15.0±6.0 µM at day 6–7).

### Relation between respiration and cytokines

Of the analyzed cytokines, IL-8 levels at day 6–7 correlated positively with both maximal ATP generating as well as maximal non-ATP generating rates of respiration i.e. OXPHOS_CI+II_ and ETS_CI+II_ (p = 0.046 r^2^ = 0.16 and p = 0.02, r^2^ = 0.22 respectively). Of the analyzed cytokines TNFα, sampled at day 1–2, displayed a weak positive correlation with the patients' severity of disease as expressed by SAPS II scores (p = 0.02, r^2^ = 0.22) and APACHE II (p = 0.03, r^2^ = 0.13).

### Difference between survivors and non-survivors

In agreement with our previous findings [8], 90-day non-survivors displayed a significantly higher ETS_CI+II_ and OXPHOS_CI+II_ respiration at day 6–7 as compared to survivors [76 (47–81) vs. 46 (43–57), p = 0.004 and 60 (41–66) vs. 41 (37–47) p = 0.015 pmol O_2_×s^−1^×10^−8^ platelets, respectively]. Among the different cytokines IL-8 demonstrated the same profile with higher values in non-survivors in samples taken at day 6–7 compared to survivors (35 (22–47) vs. 18 (13–27) pg/ml respectively, p = 0.025). None of the other cytokines differed between survivors and non-survivors.

### Markers of mitochondrial content

MtDNA copy number and CS activity were similar as compared to controls at the different time points and did not change between the different time points analyzed and also correlated to each other (r^2^ = 0.38, p<0.001). Cyt *c* was reduced by 24±5% (p = 0.03) at day 1–2 as compared to controls and then returned to similar values as controls at the subsequent time points. Cyt *c* levels also correlated to CS activity, although weaker than mtDNA (r^2^ = 0.17, p = 0.01).

### Influence of septic plasma

**Figure 3:**
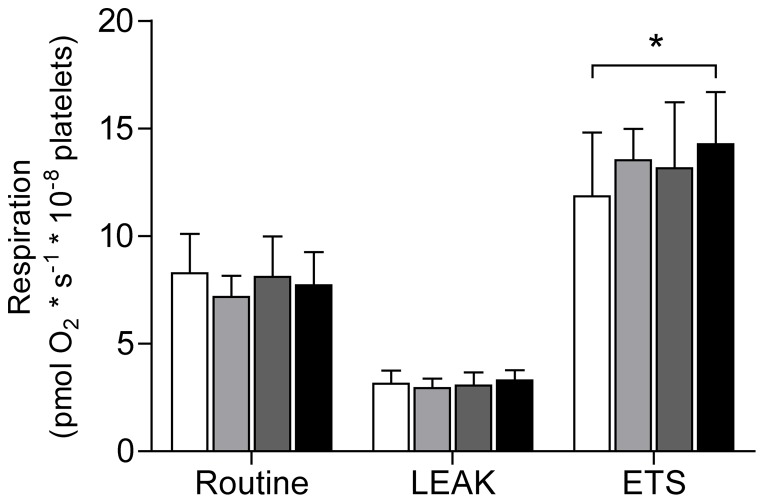
Influence of septic plasma on mitochondrial respiration. Intact platelets incubated for 1(white) or septic patients on day 1–2 (light grey), day 3–4 (dark grey) or day 6–7 (black) post admission to ICU. Stimulated to maximal respiration with FCCP. Controls n = 19, patients day 1–2 n = 18, day 3–4 and day 6–7 n = 17. Displayed as mean ± SD. * = p<0.05.

As shown in Fig. 3, platelets from healthy donors incubated in plasma from septic patients, sampled on day 6–7, displayed significantly increased maximal respiratory capacity as compared to controls (14±2.4 vs. 12±3.0 pmol O_2_×s^−1^×10^−8^ platelets, p = 0.03). Routine respiratory rate and LEAK state were similar regardless of which plasma they were exposed to.

### Effect of IL-8 on mitochondrial respiration

Platelet mitochondrial respiration remained similar as compared to controls after 24 hours of incubating platelets with IL-8 at 10 ng/ml (data not shown).

## Discussion

High-resolution respirometry performed in the O2k Oxygraph is a sensitive method of analyzing mitochondrial respiration. With the present experimental protocol we could assess whole cell mitochondrial function as well as the contribution of the individual complexes I, II, IV, V and the maximal function of the ETS with convergent electron input through both complex I+II. Accordingly this generates a comprehensive picture of the respiratory capacity of the mitochondria analysed. The evaluated mitochondrial respiratory function is also independent from the levels of markers of platelet activation since thrombin receptor agonist peptide-6 (TRAP) stimulation was not associated with an increase in respiration in our experimental setup (data not shown).

The respiratory data on the 20 patients newly recruited for the present study were in agreement with those previously presented [8]. Upon admission to the ICU, cellular mitochondrial respiration was similar to controls, in all respiratory states measured. This was followed by a progressive increase in respiration during the course of sepsis reaching an approximately 50–60% increase in all of the respiratory states measured by the end of the first week of disease. Thus, we believe that this reflects the net result of the various stimuli exerted on mitochondria coping with a supposedly increased energy demand in sepsis.

Increased plasma cytokine levels have been demonstrated in several studies of human sepsis and both individual cytokines as well as different combinations have been used as predictors for severity of disease and outcome [14], [15], [27]–[30]. In the present study, all cytokines, except for INFγ, were upregulated at the first time point measured, compared to controls, ranging from approximately 100% higher concentrations for IL-4 and 5 to several thousandfold for IL-6 and IL-10 which is in accordance with previous studies [14], [29]. After the initial peak concentration there was a steady decline at the subsequent time points but IL-10, IL-17, IL-6, IL-8, MCP-1 and TNFα remained elevated throughout the first week similar to the finding in a large multicenter study investigating the cytokine response in severe sepsis due to pneumonia [15].

Plasma NO_2_+NO_3_ levels displayed a similar, however non-significant, trend being 171% higher compared to controls upon admission to the ICU with a subsequent decrease towards control values at later time points. The levels of NO_2_+NO_3_ measured in the present study were in the same range as those previously reported [31].

Of the analyzed cytokines, IL-8 differed significantly between survivors and non-survivors in the last two time points studied. In accordance with this, IL-8 has previously been analyzed in septic patients where the levels were found to be higher in non-survivors compared to survivors [32], [33]. Another study tried to refine the prediction of outcome in septic patients and found that when combining the levels of IL-6, IL-8 and IL-10 they could demonstrate a 2-3 fold increased hazard ratio of not surviving the septic event, both early (day 3) and late (day 28) following admission [28]. This was however not replicated in the present study as neither IL-6 nor IL-10, alone or in combination with IL-8, correlated with mortality.

With regard to mitochondrial function, respiration at day 6–7 displayed a correlation with IL-8 levels at the same day of sampling but not in relation to the earlier time points. Also, as shown in our previous study [8] maximal respiratory capacity on day 6–7 were significantly higher in non-survivors compared to survivors. This finding was paralleled here with an ability of plasma, sampled from septic patient in the later stages of the disease, to induce an increased respiratory capacity in intact non-septic cells. In contrast, incubation of platelets with only IL-8 did not exert any effect on mitochondrial respiration. For the other cytokines and/or NO levels we could not detect any correlation between the increase in platelet mitochondrial respiration neither expressed as absolute values nor as the relative increase from day 1–2 to day 6–7. Both cytokines and NO act with dual mechanisms in relation to mitochondrial function in that they can provide both inhibitory as well as stimulatory effects. TNFα has been shown to inhibit OXPHOS in liver cells by subunit tyrosine phosphorylation of complex IV and to cause inhibition of complex I and II in fibrosarcoma cells [34], [35]. In contrast, mitochondrial respiration in endothelial cells was enhanced after TNFα incubation and the cells displayed increased protein content of PGC-1α, TFAM and NRF-1 as an indication of induced mitochondrial biogenesis [20]. The same phenomenon has been described for NO where multiple studies have provided evidence for its inhibitory effect of mitochondrial respiration paralleled with its ability to stimulate mitochondrial biogenesis [21], [36]–[38]. The present study does not suggest an increased mitochondrial density or protein content as an explanation for the observed elevation in oxidative capacity. This can be concluded since the increase in respiration was not accompanied by an increase in mitochondrial mass as indicated by unaltered levels of CS and mtDNA as well as the initial decrease and subsequent return of Cyt *c* levels compared to controls. Apart from a net increase in mitochondrial density, post-translational regulation of the OXPHOS system is increasingly recognized as an important modulator of respiratory capacity. Complexes of the ETS as well as a majority of the metabolic pathways in the mitochondria have been shown to be subjected to regulation by phosphorylation and dephosphorylation which, at least in part, seems to be controlled by intracellular signaling cascades triggered by extracellular receptor interactions [39]. Also, the electron transfer complexes are incorporated into larger assemblies, known as supercomplexes, or respirasomes, which are proposed to represent the optimal functional units of mitochondrial respiration and ATP production [40]. The assembly of these supercomplexes has been suggested to be regulated through cell signaling and subsequent phosphorylation [41]. The link of these regulatory steps to intra- and extracellular signaling implicates a role for cytokines as conveyers of regulatory signals. The associations found in the present study suggest a potential role for IL-8 as one such mediator. However the finding that IL-8 on its own was not able to stimulate respiration after 24 hours of incubation but plasma from septic patients was, argues against a direct short-acting effect of this cytokine and a more complex or enduring stimulus seems to be needed. The increase in respiration by plasma incubation (Fig. 3) was much smaller compared to the *in vivo* effect (Fig. 1). This would suggest that the alterations seen are to a lesser part the result of a direct stimulatory effect on the circulating platelets and to a more substantial part on the megakaryocytes during the production of platelets.

There are some limitations of the present study. Firstly, even though we have pooled the results from two patient cohorts the number of patients could still be regarded as limited. However, the two different patient cohorts displayed very similar results also when evaluated separately (data not shown). Secondly, platelets are anucleated cells and any translational change would have to take place in the megakaryocytes in the bone marrow. This together with a likely tissue diversity in mitochondrial function and regulation precludes drawing any firm conclusions regarding other tissues than the one examined. Peripheral blood immune cells have previously been investigated in septic patients [42], [43] and we have recently demonstrated a similar increase in respiration in peripheral blood immune cells [44].

In conclusion, we have demonstrated that in patients with severe sepsis and septic shock, plasma concentration of ten analyzed cytokines and NO, only IL-8 correlated with mitochondrial respiratory levels in the later phase of the disease. This suggests that cytokines as well as NO do not play a prominent role in the regulation of platelet mitochondrial respiration in sepsis. Further, the respiratory increase was not accompanied with an increase in markers of mitochondrial content, arguing for a post-translational regulation of mitochondrial respiratory capacity rather than augmented mitochondrial mass in this cell type.
