# Correction: Cytokine and Nitric Oxide Levels in Patients with Sepsis – Temporal Evolvement and Relation to Platelet Mitochondrial Respiratory Function

**DOI:** 10.1371/journal.pone.0103756

**Published:** 2014-07-22

**Authors:** The PLOS ONE Staff

## Notice of Republication

This article was republished on June 30th, 2014, due to the figures missing in the PDF version of the article. The publisher apologizes for this error.
